# Comparison of Immunotherapy versus Targeted Therapy Effectiveness in BRAF-Mutant Melanoma Patients and Use of cGAS Expression and Aneuploidy as Potential Prognostic Biomarkers

**DOI:** 10.3390/cancers16051027

**Published:** 2024-03-01

**Authors:** Zachary Garrison, Terri Clister, Eric Bleem, Elizabeth G. Berry, Rajan P. Kulkarni

**Affiliations:** 1 Department of Dermatology, Oregon Health & Science University, Portland, OR 97239, USA; 2 Knight Cancer Institute, Oregon Health & Science University, Portland, OR 97239, USA; 3 Operative Care Division, U.S. Department of Veterans Affairs Portland Health Care System, Portland, OR 97239, USA; 4 Cancer Early Detection Advanced Research Center (CEDAR), Portland, OR 97239, USA

**Keywords:** melanoma, BRAF V600E, targeted therapy, immunotherapy, progression-free survival, clinical outcome

## Abstract

**Simple Summary:**

A subset of advanced melanoma patients have a mutant BRAF protein. Two classes of therapy options are available to these patients, but it is unclear which is more effective in this population. We analyzed patient samples treated with either immunotherapy or BRAF-targeted therapy and found that those treated with immunotherapy had notably better outcomes. We further analyzed if aneuploidy or cGAS expression could act as a biomarker for how patients responded to either treatment. We found no association between aneuploidy and clinical outcomes. We observed that cGAS expression may correlate with outcome, but it appears to be a nuanced relationship and requires more study.

**Abstract:**

BRAF-mutant melanoma patients can be treated with targeted therapy or immunotherapies, and it is not clear which should be provided first. Targeted treatments do not work in up to one-third of cases, while immunotherapies may only be effective in up to 60% and come with a high risk of immune-related side effects. Determining which treatment to provide first is thus of critical importance. Recent studies suggest that chromosomal instability and aneuploidy and cyclic GMP-AMP synthase (cGAS) can act as biomarkers for cancer severity and patient outcome. Neither potential biomarker has been extensively studied in melanoma. We examined 20 BRAF-mutant melanomas treated with immunotherapy or targeted therapy and measured chromosomal aneuploidy and cGAS expression levels. Treatment type, aneuploidy, and cGAS expression were correlated with progression-free survival (PFS) in these patients. Those treated with immunotherapy first had significantly better outcomes than those treated with targeted therapy, suggesting immunotherapy should be strongly considered as the first-line therapy for patients bearing BRAF-mutant melanoma. We found that there was no correlation of aneuploidy with outcome while there was some positive correlation of cGAS levels with PFS. Further studies are needed to confirm these findings and to test other potential biomarkers.

## 1. Introduction

Among all melanoma cases, roughly half contain at least one BRAF-activating mutation. Close to 90% of those BRAF mutations are the V600E mutation [1]. The BRAF protein encodes a serine/threonine kinase in the MEK/ERK signaling pathway. Overactivation of BRAF plays a key role in the unregulated growth that drives cancer formation. Multiple BRAF-targeted therapies (i.e., vemurafenib, dabrafenib, and encorafenib) [2] have been designed to specifically treat melanomas of this genotype by limiting the mutation’s ability to affect downstream MAP kinase signaling. Unfortunately, up to a third of all patients who receive these BRAF-targeted therapies do not respond to treatment. Understanding why some patients do not respond is especially important, as there are now treatment options for those with advanced BRAF-mutant melanomas: currently either immunotherapy or targeted therapies. Immunotherapies (i.e., pembrolizumab, nivolumab, and ipilimumab) are anti-PD-1 or anti-CTLA-4 antibodies that prevent the suppression of the immune system by tumor cells. These therapies are only effective in up to 50–60% of patients, and almost 90% of patients will experience an immune-related adverse event (irAE) side effect. Determining which treatment to provide first in BRAF-mutant carrying melanoma patients is of critical importance, and there is thus a need for predictive biomarkers for these patients to help determine which therapy to use. Recent evidence suggests immunotherapy followed by targeted therapy when needed is the best choice for these patients [3]. Further understanding under what situations and for which patients this is true will have important clinical implications. With only a few studies identifying potential biomarkers for response to BRAF-targeted therapy [4,5,6], we set out to determine whether aneuploidy within the collected melanoma tumor tissue might predict the success of BRAF-targeted treatment or immunotherapy and overall disease response.

Aneuploidy, which is defined as an abnormal number of chromosomes, is found in an estimated 90% of all solid cancers [7]. Previous studies have shown a strong correlation between chromosomal instability and cancer growth and metastasis [8,9,10]. This correlation is not universal, however, and in cell lines and mouse models of aneuploidy, a lack of proliferative ability was observed, indicating aneuploidy may be protective against tumorigenic growth in certain contexts [11,12]. Within human cancers, many studies on clinical samples have found an inverse association between aneuploidy and patient survival. However, the association between aneuploidy and patient survival varied in studies that specifically evaluated melanoma patients [13]. More recent studies on melanoma patient outcomes evaluated aneuploidy and immunotherapy (anti-CTLA4, anti-PD-1, or combination treatment) and found that aneuploidy correlated with worse progression-free survival (PFS) and overall survival for metastatic melanoma patients on immunotherapy [14,15]. These studies did not evaluate patient BRAF-mutant status in their analysis, so it is unclear how aneuploidy may impact the subset of patients bearing the mutation treated with immunotherapy but does suggest aneuploidy may be a marker for response to this class of treatment. The relationship between aneuploidy and the response to BRAF-targeted therapy has not yet been reported. While a connection between aneuploidy and cancer is well documented, there is still much to be learned about the nuances of the impact of imbalanced chromosomes on cancer. We sought to explore if aneuploidy is correlated with PFS for the subset of melanoma patients with the BRAFV600E mutation treated with BRAF-targeted therapy or immunotherapy.

Another potential biomarker is cGAS (cyclic GMP-AMP synthase), an activator of Stimulator of Interferon Genes (STING), which leads to downstream production of Type I IFNs and plays an important role in immune infiltration. The cGAS/STING pathway may be closely associated with aneuploidy as the pathway can be activated by cytosolic DNA and hence can respond to chromosomal instability and aneuploidy when chromosomal DNA is segregated outside the nucleus [16]. In the past few years, cGAS has been implicated in immune response mechanisms to cancer. In pancreatic ductal adenocarcinoma, cGAS has been observed to enhance CD8^+^ infiltration in tumor cells [17]. Elevated PRMT5 (protein arginine methyltransferase 5), a component of the cGAS/STING pathway, has been correlated with prolonged survival among patients with melanoma and decreased melanoma growth in murine models [18]. While cGAS’s influence on cancer development is not fully understood, it presents an interesting biomarker for study in response to immune based cancer therapies.

Using fixed melanoma tumor samples from our institution’s biobank, we set out to determine whether BRAF-targeted therapy or immunotherapy yielded better outcomes for BRAF-mutant advanced melanoma patients. Additionally, aneuploidy or cGAS expression were correlated with treatment outcomes in these patients. We identified and collected tumor tissue blocks from twenty patients with BRAF-mutant melanomas: ten of those patients were treated first with targeted BRAF therapy, ten were treated first with either single-agent or dual immunotherapy. We began by analyzing progression-free survival, comparing the results from each treatment type. For the analysis of aneuploidy and cGAS as potential biomarkers, we analyzed chromosomes 7, 11, and 18 due to prior literature results demonstrating increased ploidy at these specific chromosomes [19,20]. Preliminary experiments with cultured melanoma cells showed noticeable increases in trisomy 11 and 18 within cells which were BRAF-targeted therapy (vemurafenib) resistant. Immunofluorescence (IF) staining of sectioned tissue slices of the melanoma blocks was used to measure cGAS expression levels on melanoma cell areas within the tissue. We predict that increased aneuploidy (in conjunction with increased cGAS expression) will be associated with increased resistance to targeted treatment and decreased PFS.

## 2. Materials and Methods

### 2.1. IRB Approvals

This study was approved by the Oregon Health and Science University (OHSU) Institutional Review Board (IRB), study #19724.

### 2.2. Sample Selection

Patient samples were screened based on the criteria of having been diagnosed with an advanced melanoma (stage IIB-IV) with a BRAFV600E mutation. After study eligibility screening, included patients were selected based on block availability and remaining tissue quantity (those with minimal or no tissue were excluded). Fixed FFPE blocks were collected from Oregon Health and Science University dermatology/pathology repositories and sectioned into 5 μm slices on polarized glass slides. 20 total patients were used in this analysis as shown in Table 1.

### 2.3. FISH Staining

**Figure 1:**
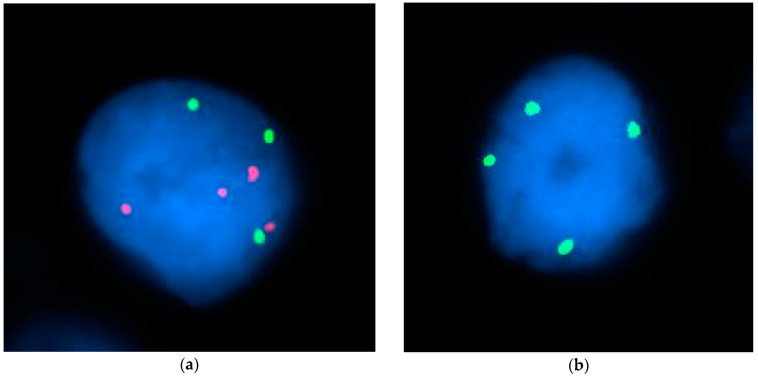
FISH staining representative images. Representative images of nuclei (blue) from FISH experiments labelling for chromosomes 7, 11, and 18. (**a**) Chromosome 7 (green), and chromosome 18 (red) FISH staining. (**b**) Chromosome 11 (green) FISH staining.

Fluorescence in situ hybridization (FISH) was completed by the Oregon Health and Science University, Knight Diagnostic Laboratory Cytogenetics core. All tissue slides submitted were stained for chromosomes 7, 11, and 18 using Vysis D7S486/CEP 7, Vysis D11Z1/CEP11, and Vysis D18Z1/CEP18 probes, respectively (Abbott Molecular). Representative images for those probes are shown below in Figure 1. Ploidy/signal counts were determined by researchers at the Knight Diagnostic Laboratory and values reported for submitted samples.

### 2.4. Immunofluorescence (IF) Staining

**Figure 2:**
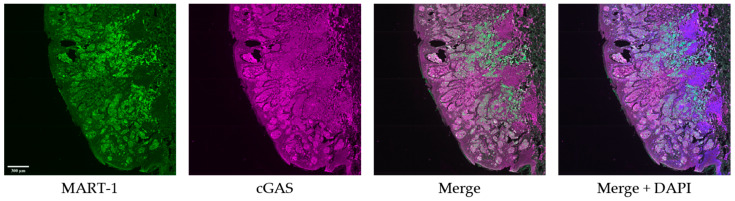
Immunofluorescence staining representative images. Representative IF images of tumor tissue co-stained with MART-1 (green), cGAS (magenta), and DAPI (blue). Scale bar = 300 µm.

Samples for IF staining were selected based on quantity of tissue remaining after FISH analysis. Tissue slides were deparaffinized by incubating in a 55 °C oven overnight followed by a one hour incubation at 65 °C. After the oven incubations, the slides were washed twice in 100% citrisolv solution for 10 min each. The slides were then washed twice in 100% ethanol solution for 10 min each. The slides were then washed for 5 min each in 95%, 70%, 50% ethanol for five minutes each. After the final ethanol wash, the slides were quickly rinsed in dH_2_O before a 10 min incubation in 1XPBS. The slides were then placed in a cup filled with 1X Citrate Buffer and placed into a pressure cooker on high for 15 min. The slides were allowed to cool for 30 min after cooking and then blocked with blocking buffer (2% BSA, 0.5 mg/mL sheared salmon DNA, 0.5% dextran sulfate in 1X PBS pH 7.4) for 30 min at room temp. After blocking, a primary antibody solution comprised of MART-1 (350 mM, biotium #BNC880668, San Francisco, CA, USA), and cGAS (350 mM, Proteintech #26416-1-AP, Rosemont, IL, USA), was added to the tissue. The slides were placed into a humidified chamber and incubated in the dark at 4 °C overnight. The following day, the slides were washed three times in 2X SSC for five minutes each. After washing the slides were fixed in 2% PFA for 15 min at room temperature. The slides were washed three times in 2X SSC for five minutes each. A secondary antibody solution was added to the slides and allowed to incubate for 45 min at room temperature in the dark. The slides were washed three times in 2X SSC for five minutes each. A DAPI nuclear stain was added to the tissue for 10 min at room temperature before it received one final set of washes in 2X SSC for five minutes. A coverslip was added to the tissue and left to harden at room temperature in the dark. Representative images are shown in Figure 2.

### 2.5. Image Collection

Slides were imaged using an Olympus spinning disk confocal microscope with a 10X objective and whole tissue slide scans were collected for each block. Individual channels were scanned separately. A spinning disk confocal microscope was selected as it was the highest power microscope available to us for this project.

### 2.6. Image Analysis

Slide scans were analyzed using Fiji (ImageJ v1.53). Using the MART-1 antibody, regions of the tissue with melanoma cells were identified and filtered out separately. cGAS expression within only MART-1 expressing cells was then selectively recorded. Expression of cGAS relative to total area of tissue and relative to MART-1 expressing area was recorded and used for comparison as some blocks varied in size significantly.

## 3. Results

### 3.1. Patients Who Received First-Line Immunotherapy Have Improved Progression-Free Survival Compared to First-Line BRAF-Targeted Therapy

**Figure 3:**
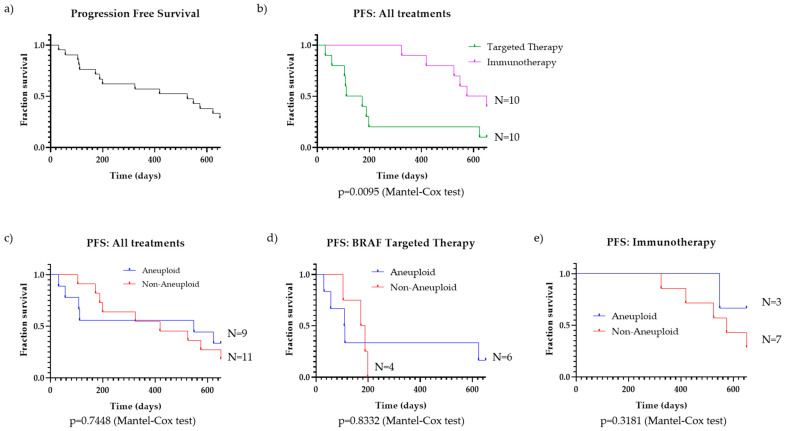
Progression-free survival (PFS) for melanoma patients. (**a**) PFS of all BRAF-mutant melanoma patients who received systemic treatment (*n* = 20); (**b**) PFS of all patients, separated by therapy group: targeted therapy (*n* = 10) and immunotherapy (*n* = 10); (**c**) PFS of aneuploid (*n* = 9) vs. non-aneuploid (*n* = 11) BRAF-mutant melanoma patients on any treatment; *p* = 0.7448 (Mantel–Cox test), (**d**) PFS of aneuploid (*n* = 6) vs. non-aneuploid (*n* = 4) BRAF-mutant melanoma patients on BRAF-targeted therapy, *p* = 0.8332 (Mantel–Cox test); (**e**) PFS of aneuploid (*n* = 3) vs. non-aneuploid (*n* = 7) patients on immunotherapy, *p* = 0.3181 (Mantel–Cox test).

We compiled data on the progression-free survival (PFS) and systemic treatment regime for the 20 patients who received systemic treatment and first looked at the outcomes collectively (Figure 3a). PFS was next compared for patients treated with BRAF-targeted therapy and those treated with immunotherapy, regardless of ploidy status (Figure 3b). As has been reported previously [3], the PFS is significantly improved for patients treated with immunotherapy compared to those treated with BRAF-targeted therapy (*n* = 10 for both treatment groups; *p* = 0.0095). These findings suggest immunotherapy should be strongly considered as the first-line therapy for BRAF-mutant melanoma patients, as clinically appropriate.

### 3.2. Certain Melanomas Exhibited Aneuploidy of Chromosomes 7, 11, and 18

We compiled the chromosome signal measurements of all twenty patients with BRAF-mutant melanoma that were analyzed using FISH and found that several melanomas exhibited aneuploidies of chromosomes 7, 11, and 18, as detailed in Table 2.

Tissues which contained an average of more than three signals per cell of any given chromosome were defined as aneuploid. Of the patient samples meeting this criterion, nine had at least one chromosome reading that averaged over three signals per cell, with chromosome 7 being the most common chromosome at elevated levels. The other 11 patient samples were measured at or below the three signals per cell threshold. Within the aneuploid cohort, the highest ploidy recorded was over six signals per cell, which corresponded to the patient with the lowest number of days before PFS (patient 1, 31 days). Only one patient (patient 6) had cells with aneuploidy measured for all three chromosomes. Intriguingly, patient 6 had the second longest PFS (623 days).

To further address the question if aneuploidy should still be considered when choosing between therapies, we segregated patients by ploidy status and compared PFS for all patients, those on BRAF-targeted therapy, and those on immunotherapy (Figure 3c–e). There was an even split in patients that were either above (N = 9) or below (N = 11) the threshold for ploidy. For all patients, those groups were segmented, and the survival curves are shown in Figure 3c. The two curves showed almost identical PFS trends throughout the duration of the analyzed time period (*p* = 0.7448).

Aneuploidy also does not correlate with melanoma PFS for BRAF-mutant melanoma patients receiving targeted therapy. Of the 10 patients who received a BRAF-targeted therapy, we compiled PFS data from the start of their treatment up to the point of disease progression and divided the cohort by aneuploid (N = 6) and non-aneuploid (N = 4) (Figure 3d). For those melanoma patients treated with BRAF-targeted therapy, there is no difference in the PFS survival curves based on ploidy status (*p* = 0.8332).

The same result is observed when comparing aneuploid (N = 3) and non-aneuploid (N = 7) patients treated with immunotherapy (Figure 3e). While there is a trend suggesting that aneuploidy is associated with improved patient outcomes, the difference in PFS is not significant (*p* = 0.3181).

After compiling survival outcomes from our patient cohort, we observed roughly equivalent PFS for patients with varying ploidy levels for all treated patients and those specifically on targeted therapy. These results suggest that ploidy levels (at least those detected through chromosomes 7, 11, and 18) do not possess prognostic capabilities for PFS outcome for patients with BRAF-mutant melanoma.

### 3.3. cGAS Expression Has a Positive Correlation with Progression-Free Survival

**Figure 4:**
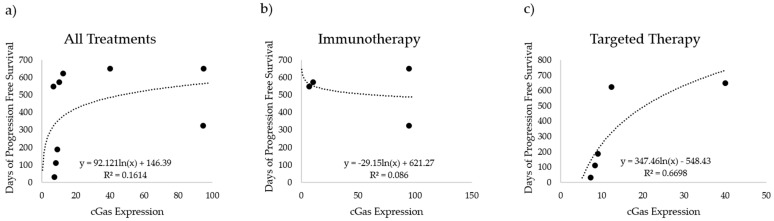
cGAS expression versus progression-free survival (PFS). cGAS expression (cGAS fluorescence/MART1 area) compared to the number of days with no progression for (**a**) all tested patients (*n* = 9), (**b**) patients treated with immunotherapy (*n* = 4), and (**c**) patients treated with targeted therapy (*n* = 5).

In addition to aneuploidy status, we also compared cGAS expression in melanoma cells to the PFS of our patient cohort. The time until disease progression is shown relative to cGAS expression in Figure 4. We compared cGAS expression with days of progression-free survival for nine patients that had sufficient remaining tissue for the analysis (four treated with immunotherapy, and five treated with BRAF-targeted therapy). When considering all nine patients, the trends observed here suggest that there is a possible positive correlation between cGAS expression in melanoma cells and PFS (Figure 4a). That trend is not present when considering cGAS and PFS for only the immunotherapy-treated patients (Figure 4b) but becomes stronger when limiting the analysis to only patients treated with targeted therapy (Figure 4c). However, the number of samples included in this cohort is limited compared to the FISH staining data. This observation contradicts the trend we had originally expected in that cGAS expression would trend similarly to ploidy levels. Additional studies with larger cohorts are needed to further explore this finding.

## 4. Discussion

Our most significant finding is that for BRAF-mutant advanced melanoma patients, first-line immunotherapy provides a better treatment outcome than first-line targeted therapy. This observation supports previously reported findings [3] but shows an even stronger difference in outcome between treatment types. It is clear from our results that immunotherapy should be strongly considered (as clinically appropriate) as the first-line therapy for patients with advanced melanoma bearing a BRAF mutation.

While we expected to find that aneuploidy and cGAS expression would show similar trends, our analysis revealed the opposite. There is no correlation between aneuploidy and PFS, but there is an association between cGAS expression and PFS in the BRAF-mutant cancer samples screened, specifically for the targeted therapy treatment cohort. Aneuploidy levels (of chromosomes 7, 11, and 18) did not demonstrate any trend for PFS for either all treated patients collectively, or for the subset of patients treated with BRAF-targeted therapy or immunotherapy. There are some important caveats to this analysis, including that we have a relatively small sample size, though even considering this, there was no trend towards PFS. Another caveat is that the three signals/cell threshold that was used to distinguish aneuploidy could be amended to be lower (hence qualifying more samples). Theoretically, anything above or below a signal threshold of two would be considered abnormal. Despite these limitations, our aneuploidy analysis is still a useful finding as it suggests that there is an additional nuance or considerations regarding how chromosomal instability can influence cancer outcomes. One possibility is the need for more than one aneuploidy, or a specific aneuploidy that is particularly tumorigenic and was not evaluated in this study. The lack of association between aneuploidy and PFS in these patients could also be attributed to the complex and contrary nature of aneuploidy and cell proliferation and immune activation or evasion. As discussed above, studies have found both anti-proliferative and tumorigenic effects of aneuploidy that depend on tumor type, degree of aneuploidy, and additional factors such as secondary mutations [10,11,12]. The paradoxical results from different aneuploidy experiments may help explain why our results differ from previous melanoma-specific studies and show no correlation between aneuploidy and PFS, given the unknown genetic factors that were outside the scope of this study. This may also explain the discrepancy between the expected cGAS expression and aneuploidy trends as there may be a more nuanced relationship between the specific type of aneuploidy and cGAS expression trends.

Additionally, the discrepancy between our results and those found in the literature may be explained by the targeted nature of this specific patient pool (patients with advanced melanomas with the BRAFV600E mutation). Other studies may have included a much more heterogenous pool of cancer types which themselves may have unique links between aneuploidy and disease progression compared to melanoma. We originally hypothesized that limiting our patient pool to BRAFV600E-bearing melanoma patients would show a stronger correlation with aneuploidy and poorer survival based on studies showing that BRAFV600E can induce aneuploidy [21,22]. However, these prior studies were performed in cell lines, and similar in vitro studies of induced aneuploidy show an anti-proliferative affect in vitro and in vivo, suggesting the level of BRAFV600E-induced aneuploidy may not be tumorigenic by itself. Another possibility is that although the BRAF mutation tends to induce greater aneuploidy, it may activate cGAS which can prevent cell proliferation by the activation of immune infiltration. This potential interplay may mask any direct association between aneuploidy and PFS for BRAF-mutant tumors.

cGAS expression did show some potential correlation with PFS. BRAF-mutant melanoma patients with elevated cGAS expression in their melanoma tumors experienced longer PFS than those with lower cGAS expression. The correlation is stronger for patients treated with targeted therapy than those treated with immunotherapy. Further studies will be necessary to confirm and refine these findings.

## 5. Conclusions

We found that immunotherapy leads to significantly better outcomes than targeted therapy in BRAF-mutant advanced melanoma patients. Further analysis found that although chromosomal instability and aneuploidy has been correlated with worse outcomes in several basic science cancer studies, in the translational patient samples we utilized, there was no correlation of the aneuploidies of chromosomes 7, 11, and 18 in BRAF-mutant melanoma with progression-free survival. We also found that there was a somewhat positive correlation of cGAS expression with PFS in these melanomas treated with targeted therapy. Further studies and additional markers are necessary to identify those biomarkers that can predict PFS and other relevant patient outcomes for BRAF-mutant melanomas and response to treatment.

The limitations of this study are largely associated with sample size. While the BRAFV600E mutation and treatment strategies targeted in this study are quite common, identifying existing, available, and viable fixed tissue blocks was challenging. As such, we were only able to retrieve 20 patient samples which limits the strength of our analysis. While we have identified interesting trends associated with cGAS expression in melanoma treatment response, our sample size limits the strength of our interpreted association.

## Figures and Tables

**Table 1:** Patient sample characteristics (na = information not available).

Patient	Gender	Age	Breslow Depth (mm)	Tissue Type	Stage	Location	Ulcerated *	Lymph Node Metastasis	Treatment
1	M	86	11.6	Primary	IIIB	Toe	Y	1	encorafenib
2	M	51	4.3	Primary	IV	Calf	Y	>1	encorafenib
3	M	72	3.0	Primary	IV	Neck	N	>1	encorafenib
4	M	74	0.4	Metastasis	IV	Groin	N	1	pembrolizumab
5	M	54	1.2	Primary	IIIA	Forearm	N	1	nivolumab
6	F	29	1.4	Primary	IIIA	Chest	N	1	trametinib/dabrafenib
7	M	72	5.1	Primary	IIIC	Buttock	Y	1	trametinib/dabrafenib
8	M	51	5.4	Primary	IIIC	Scalp	Y	>1	dabrafenib/trametinib
9	M	79	3.0	Primary	IIIC	Ear	Y	>1	enconrafenib/dabrafenib/trametinib
10	M	55	6.0	Primary	na	mid back	Y	>1	vemurafenib
11	M	45	2.9	Primary	IIB	upper back	N	>1	nivolumab
12	M	64	na	Primary	na	scalp	N	1	pembrolizumab
13	M	69	4.5	Primary	IIIC	thigh	N	>1	pembrolizumab
14	F	62	3.8	Primary	IIIC	upper foot	Y	1	nivolumab
15	F	52	6.4	Primary	IIIC	thigh	Y	>1	Nivolumab/pembrolizumab
16	M	68	4.8	Primary	IIB	scalp	Y	>1	dabrafenib
17	M	57	3.9	Primary	IIIB	upper back	N	1	nivolumab
18	M	55	1.3	Primary	II	chest	Y	na	dabrafenib
19	M	70	na	Primary	na	scalp	na	na	ipilimumab
20	M	30	1.8	Primary	IIIa	lower back	N	one	pembrolizumab

* Y—Yes, N—No, na—status not available.

**Table 2:** Chromosome signal measurements.

Sample ID	Chromosome 7 Signal	Chromosome 11 Signal	Chromosome 18 Signal
1	6.6 *	4.3 *	2
2	3.1 *	2.9	3.1 *
3	2.7	2	2.6
4	4.2 *	1.5	3.2 *
5	3	1.8	1.9
6	3.2 *	3.4 *	4.6 *
7	3.4 *	1.7	1.8
8	2.9	2.1	1.9
9	1.9	1.9	1.9
10	3.9 *	3.2 *	3.3 *
11	2.4	1.4	2.7
12	2.3	1.7	2.1
13	2.7	2.5	2.8
14	4.3 *	3.3 *	1.9
15	2.5	1.9	3
16	2.7	2.1	3.1 *
17	1.2	2	1.9
18	1.2	1.7	1.8
19	1.9	1.8	1.9
20	5.8 *	3.2 *	2.9

* denote measurements indicating aneuploidy (>3 signals).

## Data Availability

The data presented in this study are available in the article.
